# Supplementary material for: Core-Shell Nanofibers of Polyvinylidene Fluoride-based Nanocomposites as Piezoelectric Nanogenerators
Source: Polymers (Basel). 2020 Oct 13;12(10):2344. doi: 10.3390/polym12102344 (PMC7601985; doi:10.3390/polym12102344)
Supplement: Supplementary file 1 [file polymers-12-02344-s001.zip › polymers-921938-supplementary.docx]

Core-Shell Nanofibers of Polyvinylidene fluoride based Nanocomposites as Piezoelectric Nanogenerators

**Deepalekshmi Ponnamma^1^, Mariem Mohammed Chamakh^1^, Nisa Salim^2^, Nishar Hameed^2^, Mariam Al Ali AlMaadeed^2^**

^1^Center for Advanced Materials, Qatar University, P O Box 2713, Doha, Qatar.

^2^Faculty of Science, Engineering and Technology, Swinburne University of Technology, Hawthorn, Melbourne, Victoria 3122, Australia

^2^Materials Science & Technology Program (MATS), College of Arts & Sciences, Qatar University, Doha 2713, Qatar

*Corresponding Author Tel: +974 (4403) 5684; E-mail: [lekshmi_deepa@yahoo.com](mailto:lekshmi_deepa@yahoo.com) (D Ponnamma)

**Supporting Information 1: Thermal analysis**

**
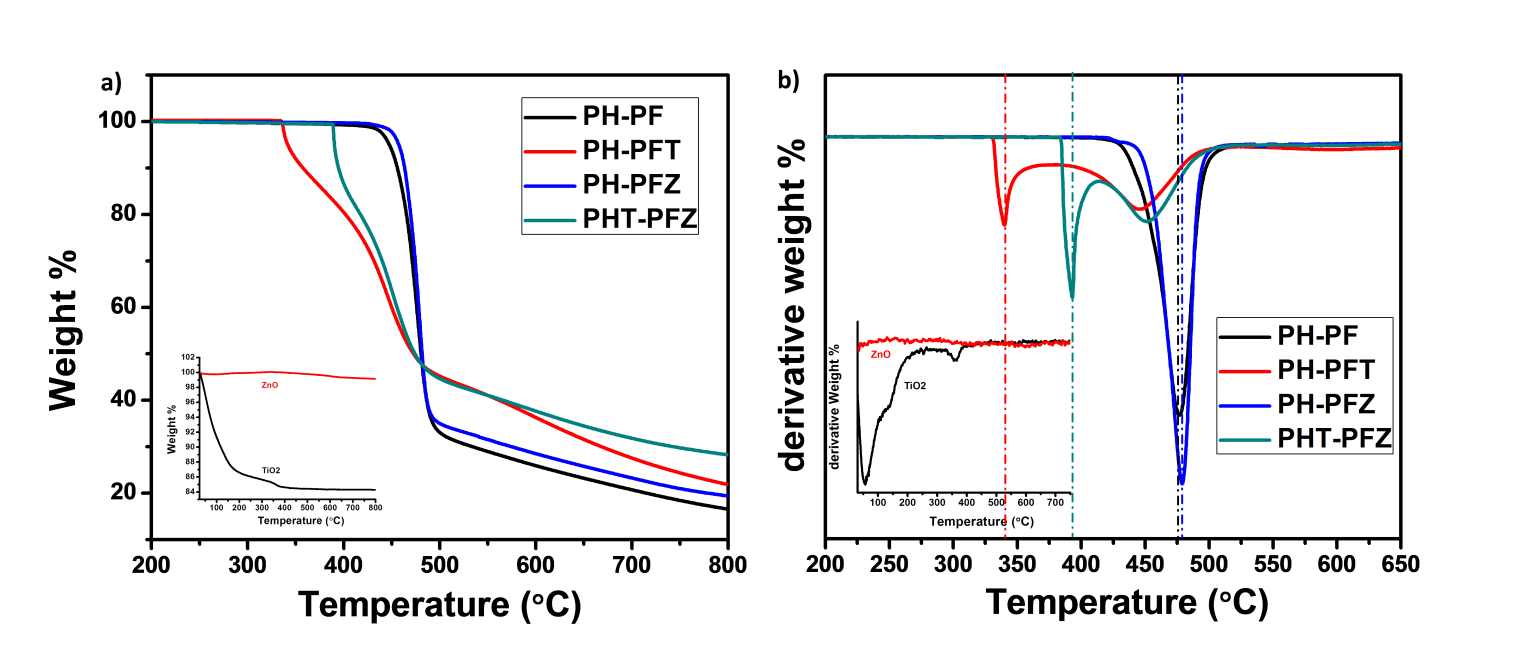
**

**Figure S1.** (**a**) Weight percentage and (**b**) derivative weight percentage for the coaxial electrospun fibers with temperature.
